# Apolipoproteins in Psoriasis: The Effect of Acitretin Treatment and UVB Phototherapy

**DOI:** 10.3390/metabo15030196

**Published:** 2025-03-12

**Authors:** Hanna Myśliwiec, Dorota Kozłowska, Katarzyna Hodun, Bartłomiej Łukaszuk, Agnieszka Owczarczyk-Saczonek, Adrian Chabowski, Iwona Flisiak

**Affiliations:** 1 Department of Dermatology and Venereology, Medical University of Bialystok, 15-540 Bialystok, Poland; 2 Department of Physiology, Medical University of Bialystok, 15-222 Bialystok, Poland; katarzyna.hodun@umb.edu.pl (K.H.); bartlomiej.lukaszuk@umb.edu.pl (B.Ł.); adrian.chabowski@umb.edu.pl (A.C.); 3 Department of Dermatology, Sexually Transmitted Diseases and Clinical Immunology, The University of Warmia and Mazury, 10-719 Olsztyn, Poland

**Keywords:** psoriasis, acitretin, phototherapy, NB-UVB, apolipoprotein, lipid disturbances

## Abstract

Background: Psoriasis is a chronic, multi-system inflammatory disease frequently associated with metabolic syndrome and lipid disturbances. Apolipoproteins, as essential regulators of lipid metabolism, may play a critical role in these metabolic abnormalities, potentially influencing disease severity and systemic inflammation. The aim of this study was to compare serum concentrations of chosen apolipoproteins in patients with psoriasis before and after treatment with acitretin or narrowband UVB (NB-UVB). Methods: This study was conducted on 39 patients with psoriasis. The concentration of nine apolipoproteins and C-reactive protein was quantified using the Bio-Plex Immunoassay Kit. Results: The serum concentrations of ApoA2, ApoC1, ApoD, ApoE, and ApoJ were higher in the acitretin group compared to the NB-UVB group before treatment, while the ApoA1/ApoA2 ratio was lower. We also observed a negative association between the Psoriasis Area and Severity Index (PASI) and ApoA1/ApoA2 ratio in the patients before the treatment. Conclusions: The results of this study confirm the presence of metabolic disturbances in psoriatic patients. The treatment with NB-UVB or acitretin did not cause any significant changes in the apolipoproteins profile. Thus, we found no detrimental impact of acitretin on the apolipoproteins profile, despite the observed rise in total cholesterol concentration after the treatment. Further research is needed to explore whether specific therapeutic approaches can modify these disturbances and potentially improve long-term cardiovascular outcomes in this population.

## 1. Introduction

Psoriasis is a chronic inflammatory disease affecting millions of people worldwide. According to K. Wang, in 2019, the global psoriasis burden included 4,622,594 incidence and 40,805,386 prevalence [1]. While the hallmark of psoriasis is the development of erythematous and scaly plaques on the skin, recent research has established that psoriasis is more than a skin condition, with significant systemic implications. Notably, psoriasis has been linked to metabolic syndrome (MS), a cluster of conditions—including central obesity, dyslipidemia, insulin resistance, and hypertension—that collectively increase the risk of cardiovascular disease and type 2 diabetes. The prevalence of MS in patients with psoriasis ranges from 20% to 50% [2]. MS is associated with an increase in the risk of diabetes mellitus, cardiovascular events, and all-cause mortality [3,4]. Patients with psoriasis have a reduced life expectancy, dying, on average, five years earlier than individuals in the general population [5].

One of the key components of MS is dyslipidemia, which includes disturbances in lipid metabolism such as elevated triglycerides (TG) and low-density lipoprotein (LDL) cholesterol, along with decreased high-density lipoprotein (HDL) cholesterol. These lipid abnormalities are not only common among patients with MS but are also frequently observed in patients with psoriasis [6,7,8]. This suggests a shared underlying pathway between psoriasis and lipid metabolism, likely mediated by chronic inflammation and immune dysregulation. Consequently, the management of psoriasis must take into account the potential impact on metabolic health, especially given the association between systemic inflammation and the worsening of cardiovascular risk profiles.

In recent years, attention has been drawn to the role of lipids and their carriers, including apolipoproteins, in the inflammatory processes associated with psoriasis. Apolipoproteins, which are key components of lipoproteins, not only participate in lipid transport and metabolism but also play signaling roles in the immune system. Their levels are often indicative of lipid homeostasis and cardiovascular risk [9]. Apolipoprotein A1 (ApoA1) and apolipoprotein B (ApoB), in particular, serve as biomarkers for cardiovascular health, with ApoA1 associated with protective HDL particles and ApoB with atherogenic LDL particles [9,10]. They have been investigated for their potential role in psoriasis, but the findings to date have been inconsistent. Until now, most research on apolipoproteins in psoriasis has focused on ApoA1 and ApoB, primarily because of their well-established roles in cardiovascular risk and lipid metabolism. These studies aim to identify biomarkers that could predict the risk of cardiovascular disease development in patients with psoriasis. In 2019, the European Society of Cardiology/European Atherosclerosis Society stated that ApoB was a more accurate marker of cardiovascular risk than low-density lipoprotein cholesterol and non-high-density lipoprotein cholesterol [9]. Additionally, among all the apolipoproteins, the serum ApoA2 level was established to be the strongest predictor of future cardiovascular events and prognosis in patients undergoing percutaneous coronary intervention [11]. In a large case-control study involving nearly 1000 patients, among 13 measured apolipoproteins, TG-carrying apolipoproteins (ApoC1, ApoC3, and ApoE) showed the strongest association with the risk of coronary heart disease [12,13]. ApoB/ApoA1 ratios are considered as good biomarkers for the prediction of cardiovascular diseases [14].

In a meta-analysis on psoriatic patients, Ramezani et al. found no significant difference between the patients and the controls in ApoA1, but the ApoB level was significantly higher in the patients than in the controls [6]. In contrast, Wang et al. revealed that psoriasis was associated with decreased serum ApoA1 and increased serum ApoB concentration. The authors included 17 studies, involving 2467 participants into their analysis. The subgroup analysis for the presence or absence of psoriatic arthritis showed that serum ApoA1 was significantly decreased in psoriasis both with and without arthritis, whereas serum ApoB was significantly increased in psoriasis with and without arthritis. [15]. Moreover, it was established that LDL cholesterol, ApoB, and lipoprotein (a) mediate the effect of psoriasis on the risk of myocardial infarction [8]. In none of those studies did the authors assess the correlation between the severity of the disease and the apolipoprotein concentration.

Acitretin, a second-generation retinoid commonly used in the treatment of moderate to severe psoriasis, is known for its effectiveness in reducing hyperkeratinization and inflammation within psoriatic plaques [16,17]. However, acitretin treatment has been associated with alterations in lipid profiles, particularly an increase in TG and cholesterol levels, which may exacerbate pre-existing lipid disturbances [18]. According to the EuroGuiDerm psoriasis guideline group, hypertriglyceridaemia is a common adverse effect of acitretin use [19]. The lipid-modifying effects of acitretin are of particular concern in patients with psoriasis, as many already exhibit dyslipidemia and other metabolic risk factors. In patients who are predisposed to or already have MS, these lipid disturbances could theoretically accelerate the progression of MS and increase cardiovascular risk. As such, evaluating the metabolic impact of acitretin is particularly relevant, and careful monitoring of lipid and glucose metabolism may help mitigate potential adverse effects associated with this treatment.

Narrowband UVB phototherapy is another well-established treatment for psoriasis, targeting inflammatory skin plaques through controlled exposure to ultraviolet B light [20,21]. This form of phototherapy is particularly effective in reducing scaling, itching, and the thickness of psoriatic lesions by slowing the rapid growth of affected skin cells. This therapy emits a specific wavelength (311–313 nm) and is often preferred over broadband UVB due to its efficacy and safety profile [22,23]. Patients generally experience a significant improvement in symptoms within several weeks, and side effects are usually limited to mild, transient erythema or itching. According to many guidelines, NB-UVB is a commonly used phototherapy option for patients with psoriasis and is an effective first-line therapy for generalized plaque psoriasis [19,20,21,24]. NB-UVB therapy is regarded as safe even in patients with metabolic disturbances, as it primarily targets skin inflammation without significant systemic effects [25].

Although advancements in biologic therapies (TNF inhibitors, IL-12/23 inhibitor, IL-17 inhibitors, and IL-23 inhibitors) have expanded treatment options for psoriasis, offering safer metabolic profiles and improved efficacy, these therapies remain inaccessible for many patients in less developed regions [19,20,21,24].

The aim of this study was to evaluate the potential associations between serum concentrations of apolipoproteins (ApoA1, ApoA2, ApoB, ApoC1, ApoC3, ApoD, ApoE, ApoH, and ApoJ) and various clinical and biochemical markers in patients with psoriasis. Specifically, we aimed to examine the relationships between these apolipoprotein levels and psoriasis severity, as measured by the Psoriasis Area and Severity Index (PASI), as well as disease duration and associated systemic inflammation, assessed by serum C-reactive protein (CRP) levels. Additionally, we have compared the effects of acitretin and NB-UVB therapy on metabolic disturbances in patients with psoriasis, focusing specifically on changes in apolipoprotein levels in serum before and after treatment.

## 2. Materials and Methods

### 2.1. Patients 

This study was conducted among 39 patients with psoriasis. Of these, 20 patients were treated with acitretin, while 19 underwent NB-UVB phototherapy.

The severity of psoriasis was estimated using the Psoriasis Area and Severity Index (PASI) [26]. Body mass index (BMI) was calculated based on weight and height. History of hypertension, heart disease, diabetes mellitus, liver disease, and the results of the laboratory tests were collected from the hospital records of the patients. Laboratory tests were measured before treatment and after about 2 months of the treatment. All psoriatic patients gave their written informed consent before enrollment in this study. The study protocol was approved by the local university bioethical committee (no. R-I-002/457/2016), and this study followed the principles of the Helsinki Declaration. Patients with chronic and acute inflammatory diseases other than psoriasis, neoplastic diseases, kidney disease, or psoriatic arthritis were excluded from this study. Patients using vitamin D (or vitamin complex) omega-3 acid supplementation were also excluded from this study. None of the patients were under dietary restriction. Patients had not been treated for psoriasis for at least 4 weeks before the enrollment.

### 2.2. Intervention

Treatment selection (acitretin or NB-UVB) was based on the severity of skin lesions (usually more severe psoriatic patients with PASI > 10 were treated with acitretin), the patient’s ability to attend phototherapy sessions regularly, and considerations for compliance with treatment protocols. Additional factors such as patient preference, access to treatment facilities, and anticipated adherence to long-term therapy were also considered in determining the optimal approach.

We adjusted the acitretin dose (0.3–0.5 mg/kg/day) to align with the patient’s clinical response and individual needs and to optimize acitretin benefit. The NB-UVB treatment schedule comprised irradiations three times a week, starting with the dose 0.020 J/cm^2^ for the II phototype and 0.025 J/cm^2^ for the III phototype. The dose was increased by approximately 10–20% per week, depending on skin type, tolerance, and clinical response to the therapy. TL-01 lamps (Cosmedico Medizintechnik GmbH, Villingen-schwenningen, Germany) were used as a source of irradiation (wavelength of 311–313 nm). The patients underwent min 10, max 20 irradiations, and their cumulative dose ranged from 0.414 to 1.278 J/cm^2^. During the entire period of irradiations, the patients were asked to apply only topical emollients on the skin.

Peripheral blood samples were collected prior to the initiation of treatment and after an overnight fasting period. After centrifugation, the serum was stored at −80 °C until analyses.

### 2.3. Determination of the Apolipoprotein Concentrations

The concentration of apolipoproteins, i.e., Apo-A1, Apo-A2, Apo-B, Apo-C1, Apo-C3, Apo-D, Apo-E, Apo-H, Apo-J, and C-reactive protein (CRP), was quantified using the Bio-Plex Immunoassay Kit (Bio-Plex ProHuman Apolipoprotein 10-Plex Assay, Bio-Rad; Warsaw, Poland). This method is based on multiplex assays with covalently coupled magnetic beads (MagPlex, Bio-Rad; Warsaw, Poland). The procedure was performed following the manufacturer’s protocol. Firstly, the serum samples were centrifuged at 1000 rpm for 15 min at 4 °C to eliminate particles. The samples were then diluted (1:50,000) in a three-step process using sample dilution buffer. The dilution factor of the standard mix was threefold. Serial dilutions of the standard were prepared according to the manufacturer’s protocol, generating an 8-point standard curve for each parameter. Subsequently, the standard, blank, control, and diluted samples were added into the corresponding wells on a 96-well microplate. Next, the capture beads were vortexed and applied to each well, and the plate was incubated for 1 h with protection from light. After that, the 96-well plate was washed three times with diluted assay buffer. The reconstituted detection antibodies were added to each well, and the assay plate was incubated a second time for 1 h. After the streptavidin–phycoerythrin solution was added and incubated for 30 min, the three washes were conducted. The beads were resuspended in each well with assay buffer, and the plate was shaken for 30 s. Finally, the microplate was immediately analyzed using the Bio-Plex 200 System (Bio-Rad Laboratories, Inc.; Hercules, CA, USA) equipped with Bio-Plex Manager Software 6.1. The analyte concentrations specified for the 8-point standard dilution set have been selected to optimize curve fitting using five-parameter logistic regression, as recommended by the manufacturer. The relative quantity of targeted molecules was indicated by the intensity of fluorescence detected on the beads. The concentration of apolipoproteins was assessed according to the appropriate standard curves generated for each apolipoprotein. Other laboratory tests (such as complete blood count, total cholesterol, HDL, LDL, and glucose) were conducted in the university hospital laboratory and were sourced from the medical records.

### 2.4. Statistical Analysis

**Figure 1:**
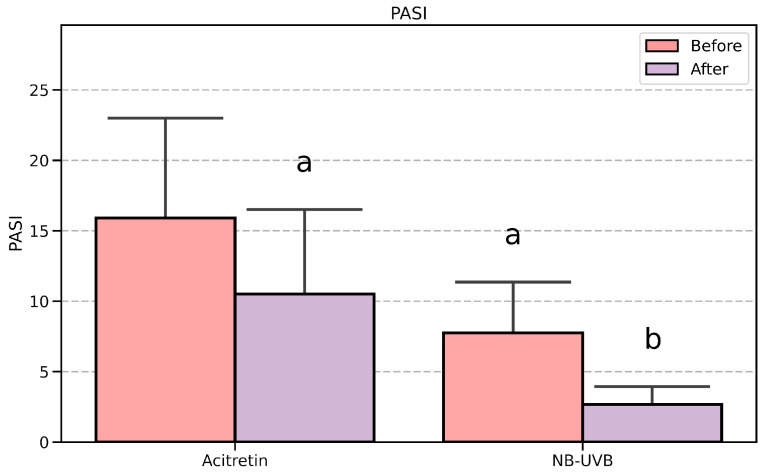
Comparison of the treatment results measured by PASI in both treatment groups. Data are presented as mean (bar height) and standard deviation (whiskers). The statistical difference is marked as follows: a—vs. Acitretin (before), *p* < 0.05, b—vs. UVB (before), *p* < 0.05, PASI–Psoriasis Area and Severity Index, NB-UVB—narrowband ultraviolet B therapy.

**Figure 2:**
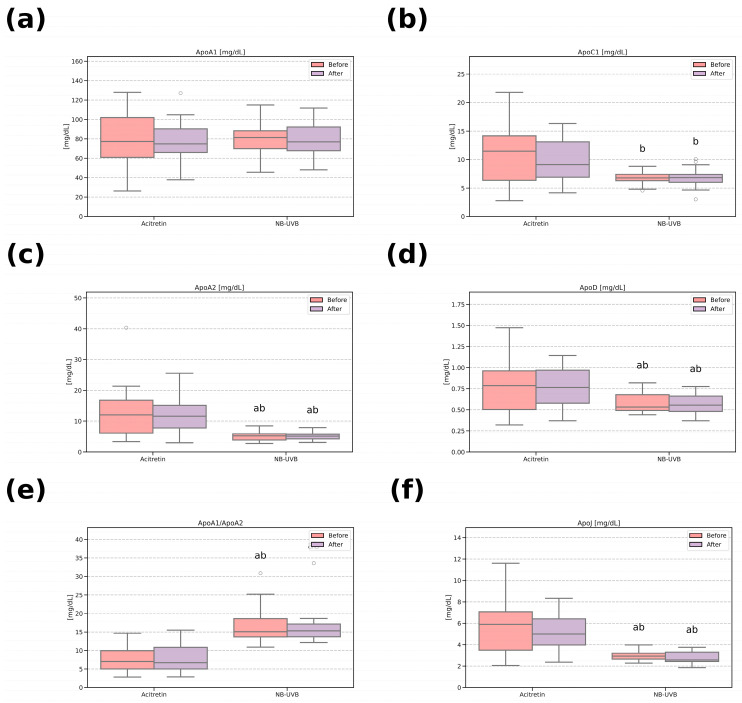
Serum concentration (mg/dL) of chosen apolipoproteins before and after treatment; comparison between the treatment groups. Data are presented as median (middle bar) and interquartile range (box), empty circles–outliers (values above 1.5*interquartile range). The statistical differences are marked as follows: a—vs. Acitretin (before), *p* < 0.05, b—vs. Acitretin (after), *p* < 0.05. (**a**) ApoA1—apolipoprotein A1, (**b**) ApoC1—apolipoprotein C1, (**c**) apolipoprotein A2, (**d**) apolipoprotein D, (**e**) apolipoprotein A1/Apolipoprotein A2 ratio, (**f**) ApoJ–apolipoprotein J. NB-UVB—narrowband ultraviolet B therapy.

**Figure 3:**
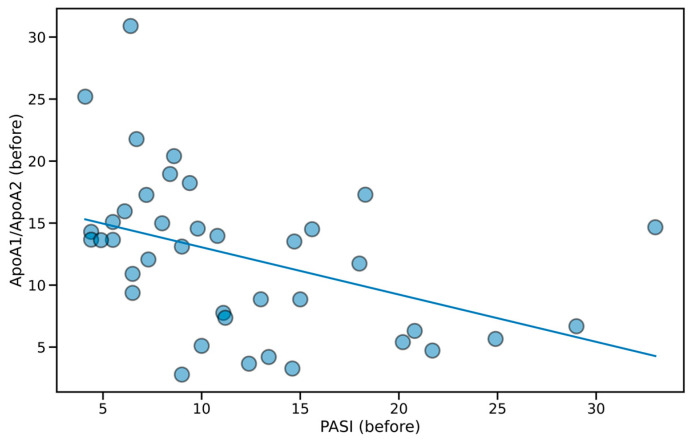
Scatterplot of PASI before treatment (both treatment groups together) vs. ApoA1/ApoA2 ratio (before treatment). Filled circles represent measurements for particular individuals. Trend line drawn based on linear regression: y = 16.87 − 0.38 × x (*p* < 0.05).

In Figure 1, bar height and whisker length represent the mean and standard deviation in the group. The data in Table 1 and Table S1 and Figure 2 are presented as the median and interquartile range (first and third quartile). On the other hand, Figure 3 contains a scatterplot where each point depicts the exact value of PASI (before) and ApoA1/ApoA2 (before) for a patient. Prior to the analysis, normality of distributions and homogeneity of variances were established using Shapiro–Wilk and Fligner–Killeen tests, respectively. Based on the results of the above, the appropriate parametric (Student’s *t*-test) or non-parametric (Wilcoxon test) methods were applied to contrast the groups. For within-group comparison (before vs. after treatment) paired tests were used, whereas between-treatment (Acitretin vs. NB-UVB) analysis was conducted with their unpaired variants. The obtained *p*-values were adjusted for multiple comparisons (Benjamini–Hochberg procedure). Categorical variables (Table 1) were analyzed using χ^2^ test with Yates continuity correction. Linear regression analysis was conducted for data presented in Figure 3. In any case, the obtained *p*-value < 0.05 was regarded as statistically significant.

## 3. Results

### 3.1. Study Population

Thirty-nine patients (6 women and 33 men), aged 23 to 78 years, with exacerbated plaque-type psoriasis were included to this study. Twenty patients were treated with acitretin. Nineteen patients underwent NB-UVB phototherapy. The median severity of the disease measured by PASI in the whole group was 9.8 (6.6–14.85). In this study group, 21 patients had mild disease (PASI ≤ 10), and 18 had moderate to severe disease (PASI > 10). From the first diagnosis, psoriasis lasted from 9 months to 66 years since the first diagnosis. There were no significant differences between the two studied groups regarding age, BMI, or disease duration. However, patients eligible for acitretin treatment presented with more severe disease at baseline compared to those eligible for NB-UVB phototherapy (median PASI 14.0 (10.82–20.35) or 6.7 (5.5–8.5), respectively). The clinical and demographic data, along with the serum concentrations of the evaluated apolipoproteins, are presented in Table 1.

### 3.2. Treatment Results

All patients in both the acitretin and NB-UVB therapy groups demonstrated a significant improvement in psoriasis lesions. In the acitretin group, the PASI score decreased from 14.0 (10.82–20.35) to 10.8 (6.0–14.22) (*p* < 0.05). In the NB-UVB group, the PASI score decreased from 6.7 (5.5–8.5) to 2.3 (1.8–3.05) (*p* < 0.05) (Figure 1).

### 3.3. Serum Concentrations of Apolipoproteins Before and After Treatment 

The serum concentrations of ApoA2, ApoC1, ApoD, ApoE, and ApoJ were significantly higher (*p* < 0.05) in the acitretin group compared to the NB-UVB group before the treatment. The ApoA1/ApoA2 ratio was lower in the acitretin group (Table S1—Supplementary Material). Further analysis of all results, both before and after treatment, revealed that despite the improvement in skin lesions and a significant reduction in the PASI score (Figure 1), no significant differences in individual apolipoproteins were observed within each treatment group (Figure 2). Post-treatment apolipoprotein concentrations remained significantly different between the two groups after the treatment (Figure 2).

In the NB-UVB group, no other significant differences were noted. However, in the acitretin group, a significant increase in total serum cholesterol concentration was observed, rising from a mean of 174.8 ± 21.94 mg/dL to 189.4 ± 33.6 mg/dL (*p* < 0.05). No significant differences were observed in other biochemical parameters before and after treatment. Despite the increase in total cholesterol, no significant changes were observed in the concentrations of the evaluated apolipoproteins, HDL, LDL, or the HDL/LDL ratio following treatment.

Additionally, our study revealed a significant negative association between the severity of the disease, as measured by PASI, and the ApoA1/ApoA2 ratio before treatment. The above is presented in Figure 3 and captured by the linear regression formula: y = 16.87 − 0.38 × x (β (slope) = −0.38, 95% CI: −0.66–0.11, *p* < 0.05), indicating that an increase in PASI score is associated with a proportional decrease in ApoA1/ApoA2 ratio (Figure 3).

Analyzing the correlations in our study, we confirmed some expected relationships: a significant positive correlation between psoriasis duration and BMI (r = 0.39, *p* < 0.05) and, additionally, between BMI and C-reactive protein (r = 0.4, *p* < 0.05). These findings suggest that, over time, patients with psoriasis tend to gain weight and experience increased systemic inflammation. 

## 4. Discussion

In this study, we analyzed the serum concentrations of nine apolipoproteins in patients with psoriasis. While limited prior research has primarily focused on apolipoproteins such as ApoA1, ApoA2, ApoB, ApoC1, ApoC3, and ApoE in psoriasis [6,15,27,28,29], our study uniquely extends this scope by also evaluating ApoD, ApoH, and ApoJ in this patient population for the first time. By exploring these less-studied apolipoproteins, we aim to broaden the understanding of lipid metabolism and its potential links to psoriasis severity and associated metabolic disturbances.

It has been well-established that psoriatic patients with moderate to severe disease (PASI > 10) are at an increased risk of developing MS, its components, or NAFLD [2,30,31,32]. Some studies have revealed a significant negative correlation between serum apolipoprotein A1 levels and systemic markers of inflammation [27,33]. It has been shown that the balance between the pro-atherogenic ApoB and the anti-atherogenic ApoA1 can help in the estimation of cardiovascular risk in psoriatic patients [34]. In our study, we did not observe the differences between serum concentrations of ApoA1 or ApoB between patients with different severities of psoriasis, but interestingly, we found that patients with more severe disease (before acitretin treatment) had higher concentrations of ApoA2 than the group of patients with mild disease before NB-UVB treatment. Moreover, some studies have linked ApoA2 directly with TG and glucose metabolism. Castellani et al. reported that overexpression of murine ApoA2 in transgenic mice resulted in the development of insulin resistance [35]. As the psoriatic population is predisposed to developing MS and diabetes, our findings may provide partial insight into this predisposition. The observed role of ApoA2 could be particularly significant in understanding these metabolic disturbances. Furthermore, previous studies demonstrated that the anti-inflammatory properties of HDL appear to be a function of the ratio of ApoA1 to ApoA2 rather than the absolute levels of each. Authors of this study concluded that ApoA2 modulates the structure and function of HDL by influencing lipolysis by hepatic lipase, analogous to the role of ApoC3 in VLDL lipolysis. Another function of ApoA2 is to modulate the anti-inflammatory/proinflammatory properties of HDL. According to the authors, both of these functions depend in part on the ratio of ApoA1 to ApoA2 [36]. Across our entire study population, linear regression analysis revealed a significant negative association between disease severity (variable PASI before) and variable ApoA1/ApoA2 ratio (β = −0.38, 95% CI: −0.66 to −0.11, *p* < 0.05). While this relation is novel, it aligns with the literature, suggesting that lipid disturbances correlate with psoriasis severity. This finding suggests that as psoriasis severity increases, the balance shifts towards a potentially more proatherogenic lipid profile. Our results potentially reflect an increased predisposition to comorbidities, including cardiovascular diseases. ApoA1 is considered anti-atherogenic, promoting cardiovascular protection, whereas ApoA2 is regarded as pro-atherogenic, potentially contributing to vascular disease. Therefore, this unbalanced ApoA1/ApoA2 ratio may provide a useful metric in assessing cardiovascular risk. This insight may facilitate timely prophylactic interventions or targeted treatments for lipid disturbances, potentially improving long-term cardiovascular health and overall longevity in psoriatic patients. Further research is needed to confirm the potential of ApoA2 or ApoA1/ApoA2 ratio as a biomarker for metabolic dysregulation in psoriasis patients.

Recent studies indicate that psoriasis patients have an increased risk of various cancers, including lymphomas and nasopharyngeal carcinomas [37,38]. Apolipoproteins can influence tumor development through different signaling pathways, either promoting or inhibiting cancer progression [39]. In our study, we identified dysregulation in the apolipoprotein A1/A2 ratio, a novel finding that has not been previously linked to cancer predisposition. In this context, its potential role warrants further investigation.

While ApoA1 and ApoB in psoriasis have been the primary focus of research due to their established roles in lipid metabolism and cardiovascular risk, other apolipoproteins, such as ApoA2, ApoC1, ApoC3, ApoD, ApoE, ApoH, and ApoJ, have not been widely studied in psoriasis. Their potential involvement in the pathophysiology of psoriasis and related metabolic disturbances remains underexplored. There are only limited data on their role in the current literature. Xiao et al. reported significantly elevated serum levels of ApoC3 and ApoE in psoriatic patients. More concerning, the highest levels were observed in young male patients (under 40 years), highlighting a potentially alarming trend in this demographic subgroup. This finding was particularly noteworthy as the patients included in the study had normal serum lipid levels [28]. Apo C3 has received remarkable attention as a key regulator of TG metabolism. In some studies, elevated plasma ApoC3 levels have been identified as independent predictors of cardiovascular mortality, regardless of fasting TG or traditional risk factors, in the general population [40]. Our findings, which show higher serum ApoC1 levels in patients with more severe psoriasis, align with the broader evidence linking apolipoproteins to cardiovascular risk. Notably, Imamura described two cases of psoriasis patients with hypertriglyceridemia and demonstrated a reduction in ApoC3 and ApoE levels following clofibrate treatment [41]. This suggests that lipid-regulating therapies may influence specific apolipoproteins, potentially mitigating associated cardiovascular risks.

ApoE and ApoJ (known also as clusterin) are associated with the development of neurodegenerative diseases, including Alzheimer’s disease [42,43], and are also important determinants of the lipid profile and cardiovascular health [44]. In our study, we observed, for the first time, higher serum concentrations of ApoJ in psoriatic patients with severe psoriasis (before acitretin treatment) compared to those with mild disease (before NB-UVB phototherapy). These findings are preliminary and warrant further investigation, particularly considering that different isoforms of these apolipoproteins may play distinct roles in the pathogenesis of Alzheimer’s disease, a mechanism that remains incompletely understood. This observation may be significant, especially in the context of reports suggesting an increased risk of Alzheimer’s disease and dementia among psoriatic patients [45,46]. Significantly higher concentrations of serum ApoD in severe psoriasis than in mild disease in the present study may have a similar meaning. ApoD is a multi-ligand, multi-functional protein that is involved lipid trafficking, food intake, inflammation, antioxidative response, and neuroprotection. Its involvement in metabolic tissues affects insulin sensitivity and glucose homeostasis in a tissue-specific manner. ApoD levels increase significantly with aging and are linked to neurodegenerative and neurological conditions, including Alzheimer’s disease [47]. Additionally, imbalances in the proportions of apolipoproteins, primarily ApoA and ApoB, have been demonstrated in dementia [48]. The role of different apolipoproteins in psoriasis and related cognitive disorders remains to be elucidated.

Apolipoprotein H (ApoH), also known as beta2-glycoprotein I, binds lipoproteins and activates lipoprotein lipases during triglyceride metabolism. Several ApoH isoforms are linked to apolipoprotein A, apolipoprotein B, HDL cholesterol, triglycerides, and total cholesterol levels. While ApoH antibodies are primarily associated with antiphospholipid syndrome and lupus anticoagulant, where they promote thrombosis [49], recent studies suggest a broader role beyond coagulation. Downregulation of ApoH has been implicated in non-thrombotic conditions, such as exacerbating fatty liver disease and disrupting gut microbiota homeostasis, pointing to its involvement in metabolic and inflammatory pathways [50]. However, in our study, we found no significant differences in serum ApoH concentrations between patients with severe and mild psoriasis, either before or after treatment. This suggests that while ApoH may play a role in metabolic and inflammatory processes, its direct contribution to psoriasis severity remains unclear.

The next intriguing aspect addressed in our study is the influence of different treatment modalities on serum apolipoprotein levels in psoriatic patients. Limited research has explored this area, with only a few studies providing detailed insights regarding methotrexate treatment of psoriasis. By examining the effects of acitretin and NB-UVB phototherapy on a broad spectrum of apolipoproteins, our investigation seeks to fill this gap. 

Wang et al. (2022) prospectively studied methotrexate’s (MTX) effects on lipid profiles and cardiovascular risk factors in psoriasis. MTX significantly reduced serum ApoB, total cholesterol, TG, HDL, lipoprotein (a), and the ApoB/ApoA1 ratio. They found that hCRP correlated with disease severity (PASI score) and the ApoB/ApoA1 ratio, while metabolic parameters were linked to blood pressure, hypertension, weight, and BMI. The authors concluded that the ApoB/ApoA1 ratio is the best predictor of CVD risk in psoriasis [27]. Similar results were found after 12 weeks of etanercept treatment, providing some potentially favorable modulation of insulin sensitivity, HDL, Apo A1, and Apo B/ApoA1 ratio [51]. Owczarczyk-Saczonek et al. did not find significant differences after 12 weeks of methotrexate treatment in serum ApoA1 levels, but ApoB levels were significantly lower after the treatment than before, indicating the beneficial effect of MTX [29]. 

According to the current literature, this is the first study investigating the influence of acitretin treatment and phototherapy on a broad range of apolipoprotein serum concentrations. Vahlquist et al. studied apolipoproteins (ApoB, ApoA1, and ApoA2) and did not find significant changes in apolipoprotein concentrations during acitretin treatment [52]. Additionally, Campalani et al. examined ApoE gene variants and demonstrated an association between the ApoE4 allele and psoriasis. However, the authors concluded that their results do not support a link between disease response to acitretin and the E2, E3, or E4 allelic variants of ApoE [53]. Acitretin can elevate triglycerides, serum lipids, and glucose, requiring monitoring before and after treatment. Hypertriglyceridemia occurs in 25–50% of patients and hypercholesterolemia in 10–30%, especially in those with risk factors like diabetes, obesity, smoking, alcohol use, or a history of hyperlipidemia [52]. Despite these effects, acitretin remains an affordable and accessible systemic therapy. A study by Sachdev on 60 psoriasis patients found that acitretin significantly increased triglycerides compared to topical treatment, though levels remained within normal upper limits [18].

In our study, we observed a significant increase in total serum cholesterol concentration following acitretin treatment. Since lipid metabolism abnormalities are closely associated with both psoriasis and cardiovascular diseases, they may act as a mediating factor in the link between psoriasis and myocardial infarction. However, in contrast to the rise in cholesterol levels, we found no significant changes in the concentrations of evaluated apolipoproteins post-treatment. Despite the observed increase in cholesterol levels, acitretin treatment appears to be safe—at least in terms of apolipoprotein concentrations. While higher cholesterol levels may raise concerns, the absence of significant changes in other lipid parameters and apolipoproteins suggests that the treatment does not cause widespread lipid disturbances. This could indicate that, under controlled conditions, acitretin remains a viable therapeutic option, especially when considering the overall risk profile in psoriasis management. Further research is needed to clarify acitretin impact on cardiovascular risk in psoriatic patients.

Narrowband UVB therapy has immunosuppressive and immunomodulatory effects, making it effective for psoriasis. It may reduce inflammatory cell communication and cytokine production, potentially via type I interferon pathways [54]. However, its metabolic impact is poorly studied, with limited evidence suggesting minimal effects on lipid metabolism.

In our study, we evaluated potential changes in specific apolipoprotein levels following treatment with acitretin, a medication known to affect lipid metabolism, and compared these results to NB-UVB therapy, which is anticipated to have minimal impact on lipid parameters. Our findings supported this hypothesis, as we observed no significant changes in serum apolipoprotein concentrations after NB-UVB therapy. These results suggest that NB-UVB does not appear to alter apolipoprotein levels, aligning with its presumed neutral effect on lipid metabolism. Duveortp et al., in a recent study, assessed the potential roles of 78 different disease mediators as biomarkers for psoriasis activity [55]. The data were analyzed to explore interactions between lesional skin mediators before and after NB-UVB treatment, as well as their correlation with serum levels. Although treatment affected the levels of a majority of mediators within the skin, no corresponding effect was observed in serum levels. Similarly to our study, the authors suggest that NB-UVB exerts its effects primarily at the skin level, with mediator levels in the skin not translating into systemic serum changes. The authors concluded that these study results strongly suggest that the effects of NB-UVB are primarily localized to the skin, with minimal systemic influence [54]. This explanation could also apply in our study; however, there is an alternative explanation: NB-UVB may have no direct effect on apolipoprotein levels or lipid metabolism. Thus, while NB-UVB effectively modulates local skin inflammation, its impact on broader metabolic pathways related to lipid profiles, based on our results, remains limited or absent.

Our study has some limitations. The sample size is relatively small, which may affect the robustness and generalizability of statistical analyses. A larger cohort would allow for more reliable subgroup comparisons and strengthen the validity of the observed correlations. Additionally, our study design was observational, limiting causal inferences regarding the relationship between apolipoprotein levels and disease severity or treatment effects. Further research with a larger population and longitudinal design is needed to confirm our findings and explore the potential clinical utility of apolipoproteins as biomarkers in psoriasis.

In conclusion, our study highlights significant differences in serum apolipoproteins between patients with severe and mild psoriasis, with disease severity correlating negatively with the ApoA1/ApoA2 ratio. This underscores the presence of more pronounced lipid disturbances in severe psoriasis. The findings of this study highlight the potential value of apolipoprotein profiling in developing increasingly personalized approaches to risk management and guiding the use of lipid-modifying medications beyond statin therapy that may further reduce the risk of cardiovascular and metabolic diseases in psoriatic patients.

While acitretin and NB-UVB treatment appeared safe concerning apolipoprotein levels, the observed increase in total cholesterol following acitretin treatment warrants further exploration. These findings emphasize the need for larger studies to assess the potential of apolipoproteins as biomarkers of cardiovascular risk of psoriasis and their implications in the management of psoriasis-related metabolic disturbances. Understanding these metabolic implications could lead to more personalized treatment plans that align dermatologic and metabolic health goals in patients with psoriasis.

## Figures and Tables

**Table 1:** Clinical and biochemical characteristics of the studied groups before treatment. Data are presented as median and interquartile range. Significant differences marked as *—*p* < 0.05. BMI–body mass index, PASI—Psoriasis Area and Severity Index, NB-UVB—narrowband ultraviolet B therapy.

	Acitretin (Baseline)	NB-UVB (Baseline)
Age [yrs]	49.5 (27.75–60.75)	49.0 (38.5–60.5)
Body weight [kg]	83.0 (74.0–88.0)	92.0 (80.0–99.5)
Height [m]	1.8 (1.7–1.8)	1.8 (1.71–1.81)
BMI [kg/m^2^]	26.6 (25.23–28.24)	28.7 (26.51–32.84)
Psoriasis duration [yrs]	14.0 (5.25–25.0)	20.0 (10.0–33.5)
PASI	14.0 (10.82–20.35)	6.7 (5.5–8.5) *
Sex (no. female/no. male)	2/18	4/15
Obesity (deg. 0, 1, 2, 3, 4)	4/13/1/1/1	3/9/3/3/1

## Data Availability

Dataset available on request from the authors.

## Supplementary Materials

The following supporting information can be downloaded at https://www.mdpi.com/article/10.3390/metabo15030196/s1. Table S1: Comparison between the serum concentration of apolipoproteins after the treatment within each treatment group.

## Abbreviations

The following abbreviations are used in this manuscript:

NB-UVB	Narrowband ultraviolet B therapy
Apo	Apolipoprotein
MS	Metabolic syndrome
TG	Triglycerides
LDL	Low-density lipoprotein cholesterol
HDL	High-density lipoprotein cholesterol
PASI	Psoriasis Area and Severity Index
CRP	C-reactive protein
BMI	Body mass index
NAFLD	Non-alcoholic fatty liver disease
MTX	Methotrexate
