# Supplementary material for: Apolipoproteins in Psoriasis: The Effect of Acitretin Treatment and UVB Phototherapy
Source: Metabolites. 2025 Mar 12;15(3):196. doi: 10.3390/metabo15030196 (PMC11944098; doi:10.3390/metabo15030196)
Supplement: Supplementary file 1 [file metabolites-15-00196-s001.zip › metabolites-3495193-supplementary.pdf]

metabolites

MDPI

Table S1. Comparison between the serum concentration of apolipoproteins after the treatment within each treatment group. Data are presented as median and interquartile range. The statistical difference marked as: a - vs. Acitretin (before), $p < 0.05$, b - vs. NB-UVB (before), $p < 0.05$, PASI—Psoriasis Area and Severity Index, CRP—C reactive protein, NB-UVB—narrowband ultraviolet B therapy.

	Acitretin (Before)	Acitretin (After)	NB–UVB (Before)	NB–UVB (After)
**PASI**	14.0 (10.82–20.35)	10.8 (6.0–14.22) a	6.7 (5.5–8.5) a	2.3 (1.8–3.05) b
**ApoA1 [mg/dL]**	77.3 (60.91–101.94)	74.8 (66.07–90.43)	81.3 (70.02–88.42)	76.9 (67.88–92.33)
**ApoA2 [mg/dL]**	12.0 (6.09–16.77)	11.6 (7.77–15.13)	5.2 (3.85–5.88) ab	5.1 (4.21–5.74) ab
**ApoB [mg/dL]**	31.9 (22.84–45.57)	31.1 (26.14–42.84)	31.9 (25.94–35.25)	31.6 (27.34–37.82)
**ApoC1 [mg/dL]**	11.5 (6.35–14.15)	9.1 (6.9–13.1)	6.8 (6.28–7.4) b	6.8 (6.0–7.37) b
**ApoC3 [mg/dL]**	1.1 (0.69–1.75)	1.3 (0.77–1.66)	0.8 (0.67–1.09)	0.8 (0.67–1.18)
**ApoD [mg/dL]**	0.8 (0.5–0.96)	0.8 (0.58–0.97)	0.5 (0.49–0.68) ab	0.6 (0.48–0.66) ab
**ApoE [mg/dL]**	0.4 (0.24–0.63)	0.4 (0.24–0.58)	0.3 (0.19–0.34)	0.2 (0.16–0.33)
**ApoH [mg/dL]**	34.4 (23.08–39.24)	32.7 (23.11–37.02)	30.2 (26.68–32.31)	29.4 (26.84–32.14)
**ApoJ [mg/dL]**	5.9 (3.49–7.08)	5.0 (3.98–6.41)	2.9 (2.65–3.2) ab	2.6 (2.43–3.31) ab
**CRP [mg/dL]**	0.5 (0.14–1.16)	0.4 (0.16–0.71)	0.2 (0.09–0.47)	0.5 (0.14–0.75)
**ApoA1/ApoA2**	7.0 (5.01–9.96)	6.7 (5.01–10.85)	15.1 (13.66–18.59) ab	15.3 (13.69–17.15) ab
**ApoB/ApoA1**	0.4 (0.31–0.58)	0.5 (0.32–0.55)	0.4 (0.31–0.5)	0.4 (0.31–0.51)
**ApoB/ApoC3**	31.4 (21.85–41.54)	31.3 (20.96–35.66)	39.2 (29.34–45.13)	37.6 (32.07–45.63)
